# Finding the 'right' GP: a qualitative study of the experiences of people with long-COVID

**DOI:** 10.3399/bjgpopen20X101143

**Published:** 2020-10-14

**Authors:** Tom Kingstone, Anna K Taylor, Catherine A O'Donnell, Helen Atherton, David N Blane, Carolyn A Chew-Graham

**Affiliations:** 1 School of Medicine, Faculty of Medicine and Health Sciences, Keele University, Keele, UK; 2 Research and Innovation Department, St George's Hospital, Midlands Partnership NHS Foundation Trust, Stafford, UK; 3 School of Medicine, Leeds Institute of Health Sciences, Faculty of Medicine and Health, University of Leeds, Leeds, UK; 4 General Practice & Primary Care, Institute of Health & Wellbeing, University of Glasgow, Glasgow, UK; 5 Unit of Academic Primary Care, Warwick Medical School, University of Warwick, Coventry, UK

**Keywords:** Primary care, primary healthcare, Covid-19, long-COVID, persistent symptoms, qualitative research, severe acute respiratory syndrome coronavirus 2, headache, chest pain, fatigue, general practice, cognitive impairment

## Abstract

**Background:**

An unknown proportion of people who had an apparently mild COVID-19 infection continue to suffer with persistent symptoms, including chest pain, shortness of breath, muscle and joint pains, headaches, cognitive impairment (‘brain fog’), and fatigue. Post-acute COVID-19 (‘long-COVID’) seems to be a multisystem disease, sometimes occurring after a mild acute illness; people struggling with these persistent symptoms refer to themselves as ‘long haulers’.

**Aim:**

To explore experiences of people with persisting symptoms following COVID-19 infection, and their views on primary care support received.

**Design & setting:**

Qualitative methodology, with semi-structured interviews to explore perspectives of people with persisting symptoms following suspected or confirmed COVID-19 infection. Participants were recruited via social media between July–August 2020.

**Method:**

Interviews were conducted by telephone or video call, digitally recorded, and transcribed with consent. Thematic analysis was conducted applying constant comparison techniques. People with experience of persisting symptoms contributed to study design and data analysis.

**Results:**

This article reports analysis of 24 interviews. The main themes include: the *‘* *hard and heavy work* *’* of enduring and managing symptoms and accessing care; living with uncertainty, helplessness and fear, particularly over whether recovery is possible; the importance of finding the 'right' GP (understanding, empathy, and support needed); and recovery and rehabilitation: what would help?

**Conclusion:**

This study will raise awareness among primary care professionals, and commissioners, of long-COVID and the range of symptoms people are experiencing. Patients require their GP to believe their symptoms and to demonstrate empathy and understanding. Ongoing support by primary care professionals during recovery and rehabilitation is crucial.

## How this fits in

It is now well recognised that a proportion of people who had an apparently mild COVID-19 infection continue to suffer with persisting and cyclical symptoms, including pain, palpitations, breathlessness, cognitive impairment, and fatigue. This is the first qualitative study in the UK to explore the perspectives of people experiencing persisting symptoms following COVID-19 infection. Participants describe the hard work of experiencing, understanding, and managing their symptoms, and the range of sources of support and help they have sought. They describe the impact on their sense of identity, and emphasise the importance of GPs believing their symptoms, and demonstrating empathy and understanding.

## Introduction

The effect of Severe Acute Respiratory Syndrome Coronavirus 2 (SARS-CoV-2) varies dramatically between individuals, from asymptomatic infection through to respiratory dysfunction and multi-organ failure. In the UK, over 130 000 people have been hospitalised with coronavirus disease (COVID-19), with 17% requiring intensive care and 26% of these people dying.^1,2^

It is becoming clear, however, that some people who had a COVID-19 infection, even those described as ‘mild’, continue to suffer from persisting or cyclical symptoms, such as chest pain and palpitations, shortness of breath, muscle and joint aches and pains, headaches, cognitive impairment (‘brain fog’), neuropathy and paraesthesia, and fatigue.^3–9^ Personal descriptions of symptoms following COVID-19 emphasise that the use of the term ‘mild’ to describe these problems does not recognise long-term sequelae or persistence of symptoms, and can be seen to diminish suffering.^10^ Similar symptoms (chronic fatigue, pain, weakness, depression, and sleep disturbance) were reported following Severe Acute Respiratory Syndrome (SARS).^11^

Post-acute COVID-19 (‘long-COVID’) is reported to be a multi-system disease sometimes occurring after a relatively mild acute illness,^12^ and people struggling with these persistent symptoms are referring to themselves as ‘long haulers’. The symptoms seem to be cyclical and variable.^13–15^ People may experience a combination of symptoms,^16,17^ including respiratory,^18^ cardiovascular,^19^ neurological,^20^ dermatological, and gastrointestinal;^16^ how long these symptoms persist is as yet unknown, and there is a limited evidence base to guide clinicians in the management of people with persistent symptoms. This uncertainty adds to patients’ concerns.^13^

These experiences indicate an urgent need to better understand the individual experience of long-COVID and help clinicians, particularly GPs, understand what is needed to help such patients in their recovery.

The aims of this qualitative study were to explore the symptoms that people who have suffered from confirmed or suspected COVID-19 infection are continuing to experience following recovery from the acute infection, and to understand their experiences of primary care support and which interventions (if any) they have found to be helpful.

## Method

Qualitative methodology, with semi-structured interviews, was used to explore the perspectives of people who continue to experience persisting symptoms following COVID-19 or suspected COVID-19 infection. Qualitative methodologies are suitable for this study due to the exploratory nature of the research questions, which seek to reveal perspectives and understandings, and interpret the experiences of people with long-COVID. Qualitative research is argued to have much to contribute to health service and health policy research by developing depth of knowledge and a more comprehensive theory base in which the ‘why?’ questions are dealt with as well as the ‘how?’ questions.^21^

This article conforms to appropriate qualitative reporting guidelines.^22^

### Recruitment of participants

People with self-reported experiences of persistent symptoms following COVID-19 infection were recruited using social media posts (on Twitter and Facebook). People were invited to contact the research team by email; when they did, they were sent an information sheet and consent form. They were asked to sign the consent form and return it to the research team.

People who agreed to participate in an interview were also invited to pass on details of the study to people they knew (snowball sampling)^23^ with similar symptoms, including to peer support groups on social media.

### Data generation

Interviews were conducted by telephone or another virtual software (such as Microsoft Teams), according to the preference of the participant. Consent was obtained by email in advance of the interviews (all conducted remotely), and reconfirmed and recorded at the start and end of the interview.

Interviews were conducted by members of the research team (CC-G, TK).

The topic guide was developed by the research team in collaboration with ‘experts by experience’ (people who were suffering with persistent symptoms) in one-to-one discussions with CC-G, and at a Clinical Commissioning Group support group in which CC-G participated. Author HA is both an expert by experience and a health service researcher. The topic guide was modified iteratively as data generation and analysis progressed in parallel. An example of prompts is given in Box 1.

Box 1 **Semi-structured topic guide prompts**

- What sort of symptoms have you had since then?
- How have these symptoms affected you?
- What sort of treatments have you taken/been offered?
- Could you tell me about any contacts with NHS 111, your GP, ambulance service, or hospital?
- Some people have described feeling very fearful about their symptoms — do you feel like this?
- Do you think that doctors know what to do with COVID-19?
- What help would you like to receive?
- What sort of symptoms have you had since then?

Demographic and symptom information (such as age, ethnicity, household composition, occupation, educational attainment, time that COVID-19 infection was experienced, whether infection was confirmed by antigen [Ag] or antibody [Ab] testing) was collected from participants in order to contextualise the data and support the description of the sample in publications.

At the end of the interview, each participant was asked if they wished to receive a summary of the findings in the form of a lay summary and/or publication(s) arising from the study. Participants were offered a shopping voucher to recompense them for their time. All participants were sent a ‘thank you’ email following the interview. A copy of the lay summary (based on initial analysis) was sent to those participants who requested it.

The digital recordings (audio only) of the interviews were transcribed by one of the research team, or a professional transcribing company.

### Data analysis

Transcripts formed the data for analysis. Thematic analysis was conducted by the whole research team using the principles of constant comparison.^24^ Thematic analysis focuses on meaning across a dataset, allowing researchers to understand collective and shared experiences.^24,25^ The research team represented a mix of professional backgrounds; this mix generated robust discussion on the interpretation of the data. CC-G analysed all transcripts; each coauthor analysed a subset; codes, themes, and alterations to the topic guide were discussed collectively and agreed. Data collection continued until the research team members were confident that saturation, at a thematic level, had been reached.^26^ A lay summary was shared with study participants, and other experts by experience, and their feedback was incorporated into the analysis. The lay summary is available as Supplementary material.

## Results

Thirty-two people responded within 72 hours of the study being advertised. A further 20 people responded in the subsequent 2 weeks. Two participants reported that they had contacted the research team after being advised of the study by a friend or colleague who had been interviewed. Not all those who initially contacted the research team subsequently returned a signed consent form. All those who returned a consent form were interviewed. The analysis of 24 interviews is presented in this paper.

Interviews lasted between 35 and 90 minutes.

The demographics of participants are presented in Table 1. None of the participants had been admitted to hospital during their initial (suspected or confirmed) COVID-19 infection.

**Table 1.** Participant characteristics

Participant number	Sex	Age	Ethnicity	Area	Education level	Work status	Otherconditions	Onset ofCOVID-19^a^	Ag test	Ab test
P1	F	20	WhiteBritish	Urban	Student	PT	Asthma	Mar	Neg	No
P2	M	22	White other	Inner city	Student	No	Hayfever	April	No	No
P3	M	60	WhiteBritish	Sub-urban	Degree	FT	Ulcerative colitis	Jan	No	No
P4	F	37	WhiteBritish	Rural	Degree	FT	Anxiety	Mar	No	No
P5	F	31	WhiteBritish	Rural	Degree	FT	Hypothyroid	Mar	Neg	No
P6	F	42	WhiteBritish	Urban	Degree	FT	Allergies	Mar	Neg	Neg
P7	F	37	WhiteBritish	Urban	Degree	FT	Hayfever	Mar	Neg	Pos
P8	M	68	WhiteBritish	Rural	Degree	Retired	Non-tuberculosis mycobacterium disease, bronchiectasis	Mar	Neg	No
P9	F	39	WhiteBritish	Urban	Degree	FT	None	Apr	Neg	No
P10	F	50	WhiteBritish	Sub-uban	Degree	FT	None	Mar	No	No
P11	F	34	Mixed heritage	Semi-rural	Degree	PT	Allergies	Mar	No	No
P12	M	67	WhiteBritish	Sub-urban	O levels	Retired	Cardiac arrhythmia	Mar	No	Pos
P13	F	30	White other	Inner city	Degree	FT	Allergies	Mar	No	Pos
P14	M	66	WhiteBritish	Rural	Degree	PT	None	Mar	Neg	No
P15	F	52	WhiteBritish	Urban	Degree	PT	Immunological defects	Mar	Neg	No
P16	F	42	WhiteBritish	Urban	Degree	PT	None	Apr	No	No
P17	F	42	WhiteBritish	Sub-urban	Degree	FT	Hay fever	Mar	Neg	Neg
P18	F	40	WhiteBritish	Sub-urban	Degree	No	Asthma, Crohns	Mar	Neg	Neg
P19	F	59	WhiteBritish	Rural	Degree	No	Hypothyroidism	Mar	No	No
P20	F	54	WhiteBritish	Sub-urban	A levels	PT	Heart surgery as infant, fibromyalgia	Mar	No	No
P21	F	42	WhiteBritish	Urban	Degree	PT	Asthma, depression	Mar	No	No
P22	F	26	WhiteBritish	Urban	Degree	PT	Asthma	Apr	Pos	Neg
P23	F	40	WhiteBritish	Sub-urban	Degree	PT	Asthma, hayfever, IBS	Mar	Neg	No
P24	F	37	WhiteBritish	Urban	Degree	PT	None	Mar	No	No

^a^All dates in 2020. FT = full-time. PT = part-time. No = none. Neg = negative result. Pos = positive result.

The four themes that will be presented are: the *‘* *hard and heavy work* *’* of enduring and managing symptoms, trying to find answers, and accessing care; living with uncertainty and fear; the importance of finding the 'right' GP; and recovery and rehabilitation: what would help?

Illustrative data are provided to support the analysis; data extracts are identified by participant number with sex (male/female) and age (years) reported in brackets for content.

### ‘Hard and heavy work’

All participants presented fluent narratives of the sequence of their symptoms, recalling the exact date of onset of their illness, with some saying that the interview provided them the first opportunity to tell their whole story.

Most participants continued the discussion after the digital recorder was turned off, emphasising their own feelings of helplessness, but also alluding to the uncertainty and helplessness that GPs had admitted to when dealing with people presenting with persisting symptoms.

#### Experiencing and enduring persistent symptoms

Participants described in detail the range, complexity, and timelines of their symptoms:

*‘But I could still feel this weight on my chest. And then after about 14, 15 days I woke up one morning and it wasn’t there. I thought, “Oh I'm done”. It was such a relief, I can remember the feeling, you know thinking, “Oh, you know I've managed to get through it” and that’s when the fatigue hit ... it was just like I’d been run over; you know I felt, gravity felt like it was applying extra on my limbs. And I couldn’t seem to manage to do anything, I stopped walking completely. And the doctor suggested, you know, post-viral fatigue and to rest, so I stopped doing anything at all. And the girls started to care for me really. And you know doing everything, cooking, cleaning, washing, shopping. And some days bed-ridden, some days you know make it to sort of breakfast to sofa, watch telly. I started to listen to the radio quite a lot because concentration was quite difficult*.’ (P10, female, 50 years)

One participant’s wife contributed to the interview, stressing the seriousness of her husband’s symptoms:

*‘He was sleeping for about 20 hours a day, 20 hours out of every 24 and he’s still sleeping now, five and half months after, he still sleeps an awful lot, sat up, not lay down, sat up, he’s just totally exhausted*.’ (P12’s wife, no demographic information)

Another participant described the impact of her symptoms on her partner and family, and how she felt they did not believe her:

*‘I think, at first, they just thought, ”Oh, for god’s sake, she’s napping again.” I feel like I constantly have to explain. I'm just exhausted and I just want to know why I'm so exhausted … I'm very conscious of my lungs because I don't want to put too much pressure on them. I know instantly when they've done too much because the pain is back, especially in the right one. It’s quite bad. I used to enjoy running, and exercising, and stuff like that. I rarely even go on walks now because I know if I walk to the end of the street, they're going to start hurting*.’ (P1, female, 20 years)

#### Managing symptoms

All participants described the lengths they had been to, trying to deal with their symptoms, while hoping that they didn’t make things worse. ’Managing’ was often something that they did alone, without seeking GP advice or support:

*‘I mean initially I started taking vitamin D. Had a joint vitamin C and zinc thing, which I didn’t take every day but I took some multivitamins, but then I was a bit unsure really … my husband’s quite anti-vitamin use … So anyway, then I took nothing for a while, and then I more recently started the vitamin D again, and I’m on B12 just because of all the burning in my feet … and a probiotic and some omega-3*.’ (P6, female, 42 years)

*‘ … so I hope the acupuncture and the massages, the deep tissue massage and the dry-needling will help, but yeah I mean you never know, what I found helpful for me with the fatigue was anti-inflammatory but also anti-histamine diet — it’s very strict, but it really helped me, so the moment I started to do it, I noticed improvements with the neurological symptoms as well …’* (P13, female, 30 years)

Most participants described how they had worked out for themselves that they needed to ‘pace’ their activities in order to conserve what little energy they had:

*‘Yeah and I really have to pace myself and I can’t do … I couldn’t do two or three household chores back to back, I have to do a chore, sit down for 15, 20 minutes and then do the next, which frustrates me, it’s like peeling potatoes, I can’t peel the carrots straight afterwards*.’ (P11, female, 34 years)

This, participants felt, was in contradiction to the advice on the NHS website *Your COVID recovery,* which stresses the importance of graded activity, and which participants described as being unhelpful:

*‘Well, one of the things that really bugged me about it was the talking about graded exercise and I’ve* *learnt* *from experience that pushing myself even a tiny bit has massive consequences* *... I did more than I should’ve done, which is still probably only 25% of what I would’ve done before I was unwell. And was absolutely floored by it. By Monday I was in bed, I couldn’t get out of bed and with chest pains. So to talk about graded exercise and not acknowledge the post-exertional malaise…’* (P19, female, 59 years)

#### Setting deadlines

A number of participants described how they had set deadlines for themselves; many focused on children returning to school or the hope of returning to work. Fear was expressed about the potential impact of not meeting the deadline set:

*‘I've got this whole September deadline in my head of, you know, when the kids go back to school, at least I’ll be able to sit quietly on my own, so if nothing else, that will be like a bonus even if I still feel rubbish. But I wonder whether once that milestone is over and if the symptoms are still there, whether I’ll start to feel hopeless again about what one expects*.’ (P16, female, 42 years)

#### Trying to find answers

Participants described how initially they sought support online from people suffering from similar symptoms, in an effort to understand and validate their symptoms, as well as seeking support and help:

*‘Finding the term ”long-haulers”, it’s about the fact that being sick is hard and heavy work*.’ (P3, male, 60 years)

Some participants, however, described how some online discussion groups had increased their anxiety and worry:

*‘Initially it was very encouraging that I wasn’t on my own, and I wasn’t making it all up and I didn’t feel like I was the only one, but the more it’s gone on, it’s scary, so I try to avoid it now, it affects my mental health, and I don’t feel like I resonate any more with some of the stuff,’* (P11, female, 34 years)

*‘ … Internet support groups, yeah on the Facebook groups that I'm on, I mean to be honest, I try not to read that group too much because it depresses me, makes me a bit anxious*.’ (P5, female, 31 years)

Seeking help had been impeded by the invisibility of symptoms, and not being believed by family and friends:

*‘Yeah well I’ll tell you what I was asked, once, one of my friends did say after quite a while, “I’m not being awful, but do you think a lot of it’s in his mind?” and I said “no”. I was quite upset about that, and then somebody else, one of my neighbours, said “How is he?” and I said “He’s still so, so tired, he’s exhausted”, and she said “Oh, but he’s a man isn’t he? Remember that!!” So, I think because you’ve not got a plaster on it, you can’t see it … ‘* (P12’s wife, no demographic information)

#### Accessing care

Some participants reported that their experiences made them feel that they were not entitled to health care:

*‘I guess I was frightened that I had an ongoing illness that was going to be chronic, and that also there was some serious pathology going on that was being missed because I guess I felt a bit like I was ineligible for health care now. I felt like I’m just going to have to live with this at home and no one will come and see me and, you know, I’m just, yeah. It was a horrible feeling actually*.’ (P6, female, 42 years)

Participants described the hard work involved in finding a GP who believed that the symptoms were real:

*‘I was initially contacting a certain GP, and that GP literally just went “you need to stay at home and rest, there’s nothing we can do”, and that frustrated me because it didn’t seem like they were being caring, it felt like I was nagging them and being a hypochondriac and that’s how I was being treated so I started contacting a different GP, in the same practice, and it’s the same outcome, they can’t do anything else but he seems to be interested and wants to know what’s going on*.’ (P11, female, 34 years)

Most participants recognised the lack of knowledge and evidence about long-COVID symptoms and management, and could understand the uncertainty faced by doctors:

*‘Well yeah, I feel like there’s a lack of knowledge. And I really wasn’t able to get any answers, I know, you know this is obviously a novel illness. But just even for **one** doctor to look into it a bit and come back to me, didn’t happen*.’ (P10, female, 50 years)

*‘Not really, just I think all the way through I found doctors that I've come into contact with are just really at a bit of a loss for it. I think at the beginning, particularly when things were going on, and not clearing up it was kind of put on me as just being a strange case ... and my GP was going, “Well, you're just weird, you know”*.’ (P5, female, 31 years)

Some participants described how interactions with GPs had compounded difficulties in how their ongoing symptoms were viewed at home:

*‘I tend to speak to different GPs every time. There was one GP who just thought it was all anxiety ... she said, ”There’s nothing wrong with your lungs. This is all anxiety. You must treat your anxiety. There’s nothing wrong with you. How are you going to manage the pandemic if you don't treat your anxiety?“ That was really upsetting because I knew I was short of breath ... I just cried and also, it didn't help because, at that stage, it wasn't known about the ongoing symptoms and my husband wasn't massively supportive at that time. When I called the GP and she said, ”There’s nothing wrong with you”, and then I told my husband what she said, that made everything even worse. I just felt very, very alone with no one to support me or talk to me ... I really did feel very, very alone and isolated at that point*.’ (P18, female, 40 years)

### Living with uncertainty and fear

#### Threat to identity

All participants described how the persistent problems following COVID had changed them; the phrase *‘compared with how I used to be’* was used by all participants:

*‘I wasn’t just fogged, I was confused. I had a very difficult encounter as a result of just being confused about things and that took a long time to resolve. I love words and I enjoy the business of communicating, and I felt that part of my life was lost. Really, I just did admin, I didn’t do anything that required clear thinking*.’ (P3, male, 60 years)

#### Fear, uncertainty, and despair

Participants vividly described their fears associated with ongoing symptoms, particularly concern that recovery may not be possible:

*‘And I’m sure I’ve got a long way to go but so I’m in a much better place than I was but yes, I was really frightened, terrified and just thought I might die on a couple of occasions ... maybe not “I’m going to die right now”, but definitely “I’m never going to get better from this” kind of feeling*.’ (P6, female, 42 years)

Participants’ accounts were pervaded by uncertainty about what was causing their symptoms and how to manage them. This uncertainty was reflected in encounters with family, friends, employers, and healthcare professionals from whom care was sought:

*‘Yes so at the moment my main fear is that obviously the virus is not … it’s new and it’s not tested and I just hope this is not something that is in my body and it will reactivate you know when I overdo any types of physical or mental activity because at the moment this is how I feel, if I overdo, in a way I need to pay back for it because the symptoms, different kinds, they come back right away*.’ (P13, female, 30 years)

All participants described feeling ignored, suggesting that the public, media and healthcare professional view of COVID was one of two extremes:

*‘So, COVID-19, it’s either a mild infection or you die? No. But no one is prepared to think about us.’* (P24, female, 37 years)

### Finding the ‘right’ GP

Participants described COVID-specific work that they had to do in order to demonstrate first that their symptoms are real, that they really are ’long-haulers‘, and then attempt to persuade healthcare professionals and provider systems to help them. However, the capacity for care was tightly controlled by the arrangement of their health professional's work, such as remote consulting:

*‘Because I’ve spoken to four different GPs throughout this. I’ve not found them very helpful, so to be honest at all. I think they would have … Yeah, I mean I haven’t found it particularly helpful. So they haven’t felt particularly interested apart from one who, there was one who I spoke to in week 13 who was kind of aghast that I hadn’t been seen, and he said, ”You need to come down now. I need to see you“, and I was a bit by that stage was like, ”Look, you’re not going to find anything“. And I think by that stage actually, to be fair to him, I didn’t give him a great history because I was a bit, you know, “No one’s interested. No one’s listening.” But it was after seeing him and him saying, you know, ”We are seeing people with ongoing symptoms but yours are very unusual”. At last, he listened*.’ (P6, female, 42 years)

Most participants wanted GPs to acknowledge their uncertainty, to feel that there was a shared journey:

*‘I have to say it was a really powerful experience speaking to the GPs ... the two more recent ones, actually just the experience of being heard and feeling like somebody got it and was being kind about it, but you know it was okay that they couldn’t do anything, I just kind of needed to know that I wasn’t losing it really and it was real what I was experiencing, I think so that was really helpful*.’ (P16, female, 42 years)

*‘She just listens a little bit more to what I'm saying and she’s much more willing to say, “Of course, we don't really know what’s going on because it’s a new virus.” She doesn't try to pretend that she understands what’s going on, which is good*.’ (P18, female, 40 years)

### What would help?

As well as being listened to, believed, and understood, people struggling with persistent symptoms expressed a need to be ‘assessed’, preferably face-to-face, with tests to investigate the cause of their ongoing symptoms:

*‘I think probably what would be good would be some sort of just having my body checked to reassure me that there’s no long term damage or some monitoring to, because we don’t know what the virus can do to your body. I suppose the things that would reassure me are things where I need my body actually checking which I don’t think you could check online, you can’t check for blood clots online, you can’t check for neurological damage online can you?’* (P4, female, 37 years)

Participants felt that a full assessment of their symptoms would need a multi-disciplinary approach:

*‘You'd have to have somewhere you could go and mention every symptom you've been having and they don't have to say exactly what’s going on because nobody knows but just to be heard, I think, is so important and have someone say, “That’s really tough what you're going through. Hopefully, it will get better but we just don't know at the moment.” What I found really useful is having some investigations. Having an echo* [echocardiogram] *made me feel very reassured … perhaps you can't do that on everybody, but I think some investigations would be good. Yeah, probably mental health, and then a cardiologist or a respiratory physician*.’ (P18, female, 40 years)

*‘What we need is a service where people can get referred to, where people listen to them and then do the screening … to screen the people who have symptoms suspicious of cardiac and respiratory issues that need further investigation. Now, that’s not to say everybody with respiratory symptoms will need further investigation … So a service whereby there are, where somebody who is able to use a commonsense approach, listens to people and says, “Well, okay, you don’t need any more”, or “you need further secondary care input as in cardiology or respiratory or whatever”, or dermatology even, you know, ‘cause there’s lots of different things. And neurology …* *‘* (P14, male, 66 years)

Participants also suggested that different phases of long-COVID require different approaches:

*‘The important thing is distinguishing between supporting recovery and managing rehab*.’ (P24, female, 37 years)

All participants emphasised the key role of the GP in supporting them at every stage.

## Discussion

### Summary

This exploratory study illustrates the range and complexity of symptoms experienced by people with persisting symptoms following suspected or confirmed COVID-19; this phenomenon is variously called ‘long-COVID’ or ‘post-acute COVID-19’, and is a new disorder,^27^ thought to affect 10% of people who had COVID-19 infection.^12^ Participants describe the hard work of experiencing, understanding, and managing their symptoms, and the range of sources of support and help they have sought: peer support, online support, complementary therapies, special diets and supplements (sometimes at great cost), and formal health care. Participants describe a change in their identity, reflecting on who they perceived themselves to be before contracting COVID-19. Participants described the difficulty of accessing care, partly due to the move to remote consulting during the COVID-19 pandemic, but also feeling that their symptoms were not believed or recognised by healthcare professionals as warranting care. All participants emphasised the importance of finding the right GP: someone who would listen to and believe them, and take their concerns seriously. Participants described how they had found ‘pacing’ key to managing symptoms, and had specific suggestions about what services to support recovery and rehabilitation are needed.

### Comparison with existing literature

There is an increasing literature on long-COVID, also known as post-acute COVID-19 infection; initial publications were by people reporting their own experiences,^3–5,10^ and highlighting the lack of responsiveness of healthcare practitioners to their problems. More recently, epidemiological data has confirmed this phenomenon,^13–15^ and there is increasing advice available to healthcare professionals about how to recognise and manage people with long-COVID^27,28^

The impact of symptoms on the identity of the sufferer resonates with Bury’s notion of ‘biographical disruption’ (the ‘shattering’ of our taken-for-granted assumptions about our bodies, ourselves, and the world in which we live), with people needing to re-think who they are and what they can do, within the family and at work.^29^ In addition, the hard work of managing the persisting symptoms, seeking care from a range of sources, echoes the literature on treatment burden — real work,^30^ which requires considerable effort from patients, their caregivers, and their extended social networks. This hard work extends to trying to access care, made difficult by the move to remote consulting. Meanwhile, participants expressed a need to be examined; the need to be reassured by rituals of a face-to-face consultation. However, people with persisting symptoms described not feeling that they are worthy of, or candidates, for health care;^31^ a position adopted because of previous negative encounters.^32^

Comparisons are emerging with Chronic Fatigue Syndrome/Myalgic Encephalomyelitis: people with symptoms struggling to make their disability visible to healthcare professionals, and to access the care and support they feel they need.^10^ As a consequence of this, people have been advised to use graded activity as a means of managing their symptoms,^33^ but then report setbacks in their symptoms. National Institute for Health and Care Excellence (NICE) has cautioned against graded activity.^28^ Most participants reported that ‘pacing’ was a helpful management strategy, particularly during the recovery phase. This has been echoed by individual long-COVID ‘activists’,^4,34^ with a call for practical guidance for GPs to help people suffering from persisting symptoms. Such guidance is gradually being published, and a number of participants in this study reported finding some documents and recommendations helpful.^35,36^

### Strengths and limitations

The authors believe this to be the first qualitative study reporting the experience of people struggling with persisting symptoms following COVID-19 infection in the UK. Participants reported feeling heard, sometimes for the first time. Participants were recruited using social media rather than through primary care, as this condition is largely undocumented; this approach supported an effective recruitment process, with identification of potential participants via Twitter proving particularly fruitful. Participants identified themselves as suffering from long-COVID; a minority had undergone investigations to exclude ongoing pathology.

Analysis was conducted by the research team, with feedback from experts by experience. Participants were predominantly female, White British, and educated to degree level. It is not known whether this reflects the population of people with long-COVID; emerging evidence for COVID-19 indicates people from Black, Asian, and Minority Ethnic groups are at higher risk. Further research regarding long-COVID is warranted. Half of the participants had not been tested for COVID-19 and, of the half that were, only one had a positive Ag test. Availability of tests was limited at the time the participants had the acute infection; furthermore, evidence indicates COVID-19 may not be detected by current tests 5–7 days after the initial onset.^37^

### Implications for practice

This study illustrates the impact of the complex problems suffered by people with long-COVID and the need for people to be believed, listened to and supported by their GP. Sharing uncertainty can be helpful to patients where symptoms are unexplained, and is certainly less harmful than symptoms being dismissed.^38^ Supporting the person to find out what might help them, and the provision of up-to-date resources for patients and clinicians, is vital rather than leaving patients to source information themselves.

So far in the COVID-19 pandemic, surveillance systems are not monitoring ill health and long-term implications of COVID-19; only deaths are reported. It is vital to improve the reporting of non-laboratory confirmed clinical cases by establishing how existing systems can be adapted or used for this purpose.Patients with post-acute or long-COVID^39^ could then be coded and monitored as appropriate. Public health bodies must also universally agree definitions of what constitutes recovery, to estimate the true burden of ill health associated with COVID-19 infection.^40^ Further research is needed to better understand recovery trajectories, to inform and improve the care of patients.^41^

The findings from this study will provide important contributions to the development of flexible, person-centred interventions for people recovering and rehabilitating from long-COVID.
